# Genomics research at Bioinformatics of Genome Regulation and Structure\ Systems Biology (BGRS\SB) conferences in Novosibirsk

**DOI:** 10.1186/s12864-019-5707-0

**Published:** 2019-05-08

**Authors:** Yuriy L. Orlov, Alex V. Kochetov, Guoliang Li, Nikolay A. Kolchanov

**Affiliations:** 1 Institute of Cytology and Genetics SB RAS, 630090 Novosibirsk, Russia; 2 Novosibirsk State University, 630090 Novosibirsk, Russia; 3 A.O.Kovalevsky Institute of Marine Biological Research of RAS, 119334 Moscow, Russia; 4 College of Informatics, Huazhong Agricultural University, Wuhan, 430070 China

In this Special Issue of *BMC Genomics* we present the studies on computational genomics discussed at BGRS\SB-2018 (Bioinformatics of Genome Regulation and Structure\Systems Biology) multi-conference (http://conf.bionet.nsc.ru/sbb2018/en/). This traditional biannual meeting on bioinformatics and systems biology in Novosibirsk Academgorodok has longest history since 1998 and wide range of special post-conference journal issues covering modern computational genomics and systems biology applications. Parallel special issues of BioMed Central journals after the BGRS conference series include manuscripts in the fields of plant biology, genomics, bioinformatics and genetics, and collated as *BMC Systems Biology*, *BMC Evolutionary Biology*, *BMC Bioinformatics*, *BMC Genetics*, *BMC Medical Genomics* and *BMC Plant Biology* Supplements this year [1–3], as well as in the past [4–7]. First special issue after BGRS\SB-2014 conference at *BMC Genomics* was published in 2014 (https://bmcgenomics.biomedcentral.com/articles/supplements/volume-15-supplement-12), and continued by Supplement issue in 2016 [4].

Note the original research paper on oscillatory dynamics in the synthesis of viral components by V.A.Likhoshvai [8] that served as excellent example of science background for next publications in the gene networks theory by the authors group. We regret to inform that outstanding scientist Dr. Vitaly A. Likhoshvai passed away February this year. His ideas on mathematical models were presented later at BMC Microbiology special issue associated with bioinformatics event - Young Scientists School “Systems Biology and Bioinformatics” (SBB’2015) [9], and evolutionary model published recently at Sci Rep journal [10], continued by Dr. Tamara M. Khlebodarova and colleagues.

Do discuss genomics researches we would also like to bring attention of the reader to the highlights of genomics and bioinformatics research at “Belyaev Readings-2017” - academician D.K. Belyaev memorial conference on genetics (http://conf.bionet.nsc.ru/belyaev100/en), which was held in Novosibirsk in 2017, in BioMed Central journal series [11–16] and Vavilov journal of genetics and breeding [17, 18].

The papers here were presented in the frames of BGRS\SB–affiliated symposium “Systems Computational Biology” (http://conf.bionet.nsc.ru/bgrssb2018/en/) and at Tenth International Young Scientists School SBB-2018 (http://conf.bionet.nsc.ru/sbb2018/en/), educational event on systems biology with own history and publication record [9, 19, 20], see also *BMC Genomics* Supplement (https://bmcgenomics.biomedcentral.com/articles/supplements/volume-16-supplement-13).

Some of the materials collated within current issue were discussed at the symposium “Biodiversity: genomics and evolution” (BioGenEvo-2018) (http://conf.bionet.nsc.ru/BioGenEvo/en/) [3].

Though genomics field is quite large, here we collected works from general computational modeling, computational approaches for human genomics and databases to applications on animal models and plant genomics. Below is a summary of the papers with some references to background publications.

Wang and coauthors [21] present method for chromosome structure analysis on cooperation of transcription factors (TF) based on integration of DNA sequences, ChIP-Seq and ChIA-PET data. ChIA-PET technology was initially designed for transcription factor binding studies [22]; it continues extends application fields including theoretical bioinformatics models [23, 24]. Chromosomal architecture has hierarchical structure from large chromosome territories to A/B compartments, topologically associated domains to chromatin loops. Chromatin loops, plays an important role in cellular functions, can bring distal regulatory elements, such as enhancers in linear DNA, to the promoters of target genes in 3D space. The authors proposed the HidPET (Hierarchical and Dynamics Analysis of TF Cooperation with ChIA-PET and ChIP-Seq Data) method for investigating the cooperation of transcription factors by the integrative analysis of available sequencing data. Based on four cancer cell datasets, the authors show association of 3D chromatin organization and gene with the hierarchical and dynamic prosperities of TFs binding.

Viktor Shamanskiy and co-authors [25] present software for mitochondrial imperfect interspersed repeats annotation. The algorithm developed is adapted to specific characteristics of mtDNA such as circularity and an excess of short repeats. The authors constructed interactive web available database ImtRDB depositing perfect and imperfect repeats positions in mtDNAs of more than 3500 Vertebrate species. The analyses of the collected data demonstrated that mtDNA imperfect repeats are usually short, associated with unfolded DNA structures and abundance of repeats is negatively associated with GC content. Somatic perturbations of mtDNA are accumulating with age, thus it is of great importance to uncover the main sources of mtDNA instability. Such instability (somatic mtDNA deletions) depends on imperfect repeats between distant mtDNA segments. The ImtRDB database (http://bioinfodbs.kantiana.ru/ImtRDB/) presents novel type of data keeping all the types of imperfect repeats in circular mtDNA. The work continues studies on tandem repeats and sequencing quality control published at *BMC Genomics* [26, 27].

Andrey Yurchenko et al. [28] present application in animal genomics - high-density genotyping on sheep breeds. The genomes of local sheep breeds are valuable sources of genomic variants for understanding mechanisms of response to adaptation and artificial selection. The authors performed a high-density genotyping and comprehensive scans for signatures of selection in the genomes from 15 local sheep breeds reared across Russia. The results demonstrated that the genomes of Russian sheep breeds contain multiple regions under putative selection containing known candidate genes related to morphology, adaptation, and domestication (e.g., KITLG, KIT, MITF, MC1R), wool quality and, growth and feed intake, reproduction and milk-related traits. This work represents the first comprehensive scan for signatures of selection in genomes of local sheep breeds from the Russia of both European and Asian origins. These gene data could be the focus of future efforts on the improvement of local and multinational breeds using the rapidly-developing plethora of contemporary genomics tools.

Daria Konina et al. [29] analyzed long noncoding RNA - LINC01420. The major part of human genome is transcribed and produces a large number of long noncoding RNAs (lncRNAs). LncRNAs are transcripts with the length more than 200 nucleotides that do not possess long open reading frames. Nowadays there are many evidences that lncRNAs play important role in the regulation of gene expression, are involved in the development of various human diseases. The function of the major part of annotated transcripts is currently unknown, whereas different lncRNAs annotations tend to have low overlap. Recent studies revealed that some lncRNAs have small open reading frames (smORFs) that produce the functional microproteins. However, the question whether the function of such genes is determined by microprotein or RNA itself or both remains open. Konina and colleagues investigated widely expressed lncRNA LINC01420, the function of which was not described at the time of the beginning of the study. The authors used reverse transcription PCR and rapid amplification of cDNA ends (RACE) analysis to determine the structure of the LINC01420 transcript. LINC01420 has two isoforms that differ in length of the last exon and are localized predominantly in the cytoplasm. D’Lima et al. [30] found smORF in the first exon of the LINC01420 gene. This smORF produces functional microprotein named non-annotated P-body dissociating polypeptide (NoBody). However, Konina and colleagues [29] provide here new facts about LINC01420 transcript and its function.

Larisa Fedoseeva et al. [31] discuss the differences in brain stem transcriptional profiling in rat models. Essential (primary) hypertension is a widely spread disease with underlying genetic predispositions, many of which still remain unknown [32]. The development of essential hypertension is associated with a wide range of mechanisms. The brain stem neurons are essential for the homeostatic regulation of arterial pressure as they control baroreflex and sympathetic nerve activity. The ISIAH (Inherited Stress Induced Arterial Hypertension) rats reproduce the human stress-sensitive hypertensive disease with predominant activation of the neuroendocrine hypothalamic-pituitary-adrenal and sympathetic adrenal axes. RNA-Seq analysis of the brain stems from the hypertensive ISIAH and normotensive control WAG (Wistar Albino Glaxo) rats was performed to identify the differentially expressed genes and the main central mechanisms (biological processes and metabolic pathways) contributing to the hypertensive state in the ISIAH rats. The authors revealed set of differentially expressed genes and discussed their association with hypertension and blood pressure regulation, as well as association with central nervous system diseases. This study also continues research on transcriptome profiling on ISIAH rats published at BGRS special issues in 2016 [33, 34].

Nikita Ivanisenko et al. [35] discuss the role of c-FLIP/NEMO interaction in the CD95 network via rational design of molecular probes. The authors analyze the molecular interactions orchestrating death receptor signaling networks. CD95/Fas is a member of the DR family, which is a subfamily of the tumor necrosis factor receptor superfamily. Activation of CD95 initiates the extrinsic apoptosis pathway. The CD95-induced apoptotic signal is mediated via the formation of a death-inducing signaling complex (DISC), which comprises CD95, FADD, procaspases-8, − 10 and c-FLIPs (cellular FLICE-like inhibitory proteins). The DISC serves as a central platform for procaspase-8 activation, which subsequently initiates an apoptotic cascade. Recently, it has been demonstrated that, at the DISC, procaspase-8 proteins form so-called DED (Death Effector Domain) chains or filaments via interactions of their DED motifs. NF-κB activation is mediated via activation of the IKK complex containing the regulatory subunit NEMO (nuclear factor (NF)-κB essential modulator). c-FLIP proteins have been reported to play an important role in NF-κB induction and, in particular, in CD95-mediated NF-κB induction. The authors created the homology model of the c-FLIP/NEMO complex using the reported structure of the v-FLIP/NEMO complex, and rationally designed peptides targeting this complex. Further insight into the function of c-FLIP/NEMO complex was provided by the analysis of evolutionary conservation of interacting regions which demonstrated that this interaction is common in distinct mammalian species. These findings provide new opportunities for the design of new specific anti-cancer therapies based on the inhibition of death receptor-mediated anti-apoptosis pathways. Protein modeling research of the same group on SOD1 was published last year at BMC Structural Biology as selected article from Belyaev Conference 2017 [36].

The next group of papers in dedicated to plant genetics. Rozanova et al. [37] focused on identification of SNPs associated with barley resistance to novel isolates of *Pyrenophora teres F. teres*. Net blotch caused by *Pyrenophora/Drechslera teres* is a major foliar disease of barley, resulting in 40% yield losses of susceptible cultivars. Rozanova et al. detected seven genomic regions in barley associated with the resistance to four novel *Pyrenophora teres F. teres* isolates, including one novel isolate-specific locus. The significant SNPs detected in this study can be converted to convenient PCR-markers for accelerated breeding of resistant barley cultivars. Note the research of the authors group on SNP analysis in barley on microchips [38].

Konstantin Kozlov et al. [39] present regression models for plant genomics - flowering time estimation in wild chickpea. This work is published as BGRS\SB-2018 Supplement to *BMC Plant Biology* issue. Accurate prediction of crop flowering time is required for reaching maximal farm efficiency. Several models developed to accomplish this goal are based on deep knowledge of plant phenology, requiring large investment for every individual crop or new variety. Cultivars of chickpea, *Cicer arietanum*, are currently being improved by introgressing wild *C. reticulatum* biodiversity with very different flowering time requirements. The authors built a novel model for flowering time of wild chickpeas collected at 21 different sites in Turkey and grown in 4 distinct environmental conditions over several different years and seasons. Kozlov and co-authors propose a general approach, in which the analytic forms of dependence of flowering time on climatic parameters, their regression coefficients, and a set of predictors are inferred automatically by stochastic minimization of the deviation of the model output from data. They also identified several genomic markers in chickpea associated with different reactions to climatic factor changes.

We invite our readers worldwide to attend our next events - Systems Biology and Bioinformatics Young Scientists School SBB-2019 which will be held in Novosibirsk, and Vavilov Society Congress - 2019 in St. Petersburg, Russia, in summer 2019. Genomics field will be highlighted at VII Congress of Vavilov Society of Geneticists and Breeders organized by of Saint Petersburg State University 18–22 June 2019 (https://events.spbu.ru/events/genetic-selection-2019). Soon after, 24–29 June 2019, 5th International scientific conference “Plant genetics, genomics, bioinformatics and biotechnology” (PlantGen2019) will be organized in Novosibirsk, Russia (http://conf.bionet.nsc.ru/plantgen2019/en/) focusing on plant genomics field. Next BGRS\SB-2020 event is scheduled to June 2020 in Novosibirsk, Russia.
